# NAFLD and MAFLD independently increase the risk of major adverse cardiovascular events (MACE): a 20-year longitudinal follow-up study from regional Australia

**DOI:** 10.1007/s12072-024-10706-1

**Published:** 2024-07-15

**Authors:** Karl Vaz, William Kemp, Ammar Majeed, John Lubel, Dianna J. Magliano, Kristen M. Glenister, Lisa Bourke, David Simmons, Stuart K. Roberts

**Affiliations:** 1 Department of Gastroenterology and Hepatology, Alfred Health, Ground Floor Alfred Centre 55 Commercial Road, VIC 3004, Melbourne, Australia; 2 Central Clinical School, Monash University, Melbourne, Australia; 3 Diabetes and Population Health, Baker Heart and Diabetes Institute, Melbourne, Australia; 4 Department of Rural Health, University of Melbourne, Melbourne, Australia; 5 Macarthur Clinical School, School of Medicine, Western Sydney University, Penrith, Australia

**Keywords:** NAFLD, MAFLD, MACE, Cardiovascular disease, Atrial fibrillation

## Abstract

**Background and aims:**

The association between fatty liver disease (FLD) and cardiovascular disease (CVD) in an Australian context has yet to be defined. The primary aim of this study was to investigate the association between FLD and 3-point major adverse cardiovascular events (MACE).

**Methods:**

This was a longitudinal follow-up study of a randomly sampled adult cohort from regional Australia between 2001 and 2003. Baseline covariates included demographic details, anthropometry, health and lifestyle data, and laboratory tests. Non-alcoholic fatty liver disease (NAFLD) and metabolic-(dysfunction) associated fatty liver disease (MAFLD) were diagnosed in participants with fatty liver index (FLI) ≥ 60 and meeting other standard criteria. ICD-10 codes were used to define clinical outcomes linked to hospitalisations. Three-point MACE defined as non-fatal myocardial infarction (MI) and cerebrovascular accident (CVA) and CVD death.

**Results:**

In total, 1324 and 1444 participants met inclusion criteria for NAFLD and MAFLD analysis, respectively. Over 23,577 and 25,469 person-years follow-up, NAFLD and MAFLD were independent predictors for 3-point MACE, adjusting for demographic covariates and known cardiometabolic risk factors, whilst considering non-CVD death as a competing event (NAFLD: sub-hazard ratio [sHR] 1.56, 95% confidence interval [CI 1.12–2.19]; MAFLD: sHR 1.51, 95% CI 1.11–2.06). The results held true on several sensitivity analyses.

**Conclusions:**

Both forms of FLD increase the risk for CVD independent of traditional cardiometabolic risk factors.

**Supplementary Information:**

The online version contains supplementary material available at 10.1007/s12072-024-10706-1.

## Introduction

Non-alcoholic fatty liver disease (NAFLD), and the revised term metabolic-(dysfunction) associated fatty liver disease (MAFLD), share key pathophysiological drivers with cardiovascular diseases (CVD), in particular insulin resistance [1]. Natural history studies have consistently demonstrated CVD to be one of the foremost causes of death in those with fatty liver disease (FLD), significantly outweighing death from advanced chronic liver disease [2, 3]. As such, there is debate about expanding the diagnostic criteria for the metabolic syndrome (MetSyn) to incorporate FLD [4].

CVD accounted for two of the top five leading causes of death amongst Australians since 1968, with death due to ischemic heart disease (IHD) consistently the principal cause over this time [5]. In 2021–22, 14.3 billion Australian dollars—close to 10% of total health care expenditure in Australia—was attributable to CVD [6]. This places a significant burden on the healthcare system, which is likely to increase with an aging population [7].

We have previously demonstrated the age- and sex-standardized prevalence of NAFLD to have increased over a 15-year period in regional Victoria, in parallel to generalized obesity [8]. This may influence the CVD prevalence into the future. However, to date, there has not been a population-based study determining the impact of FLD on incident CVD in Australia. Further, studies conducted internationally have failed to unequivocally conclude whether FLD increases the risk of CVD, in particular, fatal CVD [9–11].

This study aimed to determine if NAFLD and/or MAFLD independently increased the risk of CVD in a cohort from regional Victoria.

## Methods

### Study cohort

This is a longitudinal follow-up study from the Crossroads I (CR-1) study conducted in eight major towns in northeast regional Victoria, Australia, between June 2001 and February 2003. The methodology of this study has been previously described in detail [12]. To summarize, households were randomly selected from residential address lists and attended in-person by trained data collectors. Households were selected 1:1 from the two main regional centers and six surrounding rural shires. From each household, all residents aged ≥ 16 years were invited to complete a health questionnaire, with one adult (≥ 18 years old) from participating households invited to enter a clinic sub-study, the Crossroads Undiagnosed Disease Study (CUDS). CUDS collected comprehensive data on demographic details, anthropometry, blood pressure, and more detailed health, diet, and lifestyle questionnaires, including information on alcohol consumption and medication use. Laboratory tests were conducted including full blood examination, biochemistry, liver function tests, fasting lipid profile, fasting glucose, glycosylated hemoglobin (HbA1c) and a urine sample. Further, all people without known diabetes undertook an oral glucose tolerance test. Women who were pregnant were also excluded from undertaking an OGGT. In total, n = 1454 participated in CUDS.

### Study outcome

The primary outcome for this study is to investigate if there is a difference in 3-point major adverse cardiovascular events (MACE) in those with NAFLD and MAFLD compared to those without fatty liver disease (FLD). The secondary outcomes are to determine differences in incidence of fatal CVD, 5-point MACE and atrial fibrillation (AF) in those with and without NAFLD and MAFLD, and estimate incidence of myocardial infarction (MI), cerebrovascular accident (CVA) (either hemorrhagic or ischemic), congestive cardiac failure (CCF) and unstable angina (UA) in those with FLD.

### Data linkage

Longitudinal outcomes were acquired through the Centre for Victorian Data Linkage (CVDL), which is the custodian for datasets on emergency department presentation, hospital admission, and deaths registered in the state of Victoria, Australia. Data availability for emergency department presentation and death registry was from entry into CUDS, whilst hospital admission data was only available from inception of that dataset (1st July 2007). Underlying cause of death (COD) was also obtained through the Australian Bureau of Statistics. Participants were censored at first occurrence of relevant outcome or 31st October 2022, the final date for data linkage.

### Study definitions

NAFLD was diagnosed in those with a Fatty Liver Index (FLI) ≥ 60 [13] in the absence of excessive alcohol consumption (≥ 210/140 g per week in men/women) and chronic viral hepatitis or alternate chronic liver disease (per self-report) [14]. MAFLD was diagnosed in those with FLI ≥ 60 and additional metabolic criteria (overweight/obesity with ethnicity-specific cut-offs for Asians, type 2 diabetes mellitus [T2DM] and/or ‘metabolic dysfunction’), irrespective of alternate cause of liver disease, per the original consensus statement [15] and as endorsed by the Asian Pacific Association for the Study of the Liver (APASL) [16].

MetSyn was diagnosed according to the harmonized criteria agreed upon by a conglomerate of expert international societies [17] as occurring in anyone meeting 3 out of the following 5 criteria: elevated waist circumference, elevated triglycerides, reduced high-density lipoprotein cholesterol (HDL-C), elevated blood pressure and elevated fasting glucose. T2DM was considered in those self-reporting existing diagnosis on administered health questionnaire or as a new diagnosis in those meeting World Health Organization and American Diabetes Association criteria [12, 18]. Dyslipidemia was defined according to lipid parameters as per the Australian Institute of Health and Welfare [19].

Baseline prevalent AF and MACE were coded per health questionnaires. Prevalent MACE included non-fatal MI, CVA, CCF and coronary revascularisation (percutaneous coronary intervention or coronary artery bypass grafting). Three-point MACE included non-fatal MI, non-fatal CVA and death due to CVD, whilst 5-point MACE also included CCF and UA [20]. Events were only considered once (e.g., if a participant had an MI and subsequent CVA, they were coded as having 3-point MACE at time of MI and censored thereafter). CVD-related death, 3- and 5-point MACE, and AF were coded according to International Statistical Classification of Disease and Related Health Problems, 10th revision (ICD-10) manual (Supplementary Table 1) and obtained through the CVDL datasets as noted above, including both primary and secondary reasons for hospitalization.

Elevated alanine aminotransferase (ALT) was considered in those with ALT ≥ 1.5 × the upper limit of normal according to gender (male 30 U/L; female 19 U/L).

Participants were excluded from analysis if there was insufficient data to evaluate FLI and further were excluded from the NAFLD analysis if they had FLI ≥ 60 and a history of excessive alcohol consumption or alternate chronic liver disease.

### Statistical analysis

Categorical data are presented as frequency (percentage), with between-group differences calculated using Pearson chi-squared test or Fisher’s exact test, where appropriate. Continuous covariates are presented as median with interquartile range (IQR] or mean with standard deviation (SD) following normality assessment, with Mann–Whitney *U* test and independent samples *t*-test utilized to test hypotheses. Incidence rates are presented per 1000 person-years, with group comparisons using incidence rate ratios (IRR), or mortality rate ratio (MRR) for death. Cox proportional hazards regression analysis was used to establish if NAFLD and MAFLD were predictors of outcome on both univariate and multivariate models. Non-CVD deaths were considered as a competing risk on Cox models, except for AF whereby all-cause mortality was considered a competing risk. Results are presented as sub-hazard ratios (sHR) with 95% confidence interval (CI). Multivariate models were adjusted *a priori* according to demographic, lifestyle and clinical risk factors known to influence the outcome of interest [21]. In detail, Model 1 adjusted for age, gender (male as reference) and education (dichotomized with reference those who had not completed secondary school or above), Model 2 adjusted for Model 1 along with smoking status (non-smoker as reference) and diet adequacy (according to sufficient consumption of fruit and vegetables per day according to Australian guidelines [22]), and Model 3 adjusted for Model 2 as well as T2DM, hypertension, dyslipidaemia and baseline prevalent MACE. Models for AF were the same, except Model 2 was also corrected for excessive alcohol consumption and Model 3 corrected for excessive alcohol consumption and baseline prevalent AF [23]. Sensitivity analyses were conducted excluding those with baseline MACE (and baseline AF for Models investigating AF as outcome), assessing those with FLD versus those with definitively no steatosis (FLI < 30), and only including codes related to primary reason for hospitalization for longitudinal outcomes. A two-tailed *p* value < 0.05 is considered statistically significant. All analyses were conducted using Stata/IC version 16.1 for Windows (StataCorp, Texas, USA).

### Ethics

The Crossroads study was approved by the Goulburn Valley Health Human Research Ethics Committee (GCH-3/99), whilst the current follow-up study has been approved by the Alfred Health Ethics Committee (project 310/22).

## Results

Following defined exclusions, a total of *n* = 1324 and *n* = 1444 participants had evaluable data for NAFLD and MAFLD analysis, with a prevalence of 35.4% (*n* = 469) and 40.7% (*n* = 588), respectively.

Compared to those without FLD, participants with either form of FLD were more likely to be male, older, smokers or ex-smokers, overweight or obese, have MetSyn and its individual components, prevalent MACE, and be taking medication for diabetes and hypertension, but not aspirin (Table 1). Furthermore, fasting levels of glucose, HbA1c, total cholesterol, low-density lipoprotein cholesterol (LDL-C), and triglycerides were higher in those with FLD than those without, whilst HDL-C was lower. There was no difference in prevalence of excessive alcohol consumption or viral hepatitis in those with and without MAFLD.

**Table 1:** Baseline demographic, clinical and laboratory covariates according to fatty liver disease diagnosis

Variable	Non-NAFLD (*n* = 855)	NAFLD (*n* = 469)	*p*-value	Non-MAFLD (*n* = 856)	MAFLD (*n* = 588)	*p*-value
Age, years	50 (38.3–65.2)	53.7 (42.9–65.8)	0.001	50 (38.3–65.2)	54.4 (44.3–66.5)	< 0.001
Male gender	294 (34.39)	265 (56.50)	< 0.001	295 (34.46)	338 (57.48)	< 0.001
Ethnic background White Asian Indigenous Other	833 (97.43) 10 (1.17) 4 (0.47) 8 (0.94)	456 (97.44) 5 (1.07) 4 (0.85) 3 (0.64)	0.779	834 (97.43) 10 (1.17) 4 (0.47) 8 (0.93)	573 (97.78) 5 (0.85) 5 (0.85) 3 (0.51)	0.576
Private health insurance	428 (50.06)	223 (47.55)	0.382	429 (50.12)	270 (45.92)	0.117
Education secondary school and beyond	433 (50.82)	197 (42.27)	0.003	434 (50.88)	230 (39.38)	< 0.001
BMI, kg/m^2^	24.98 (± 2.96)	32.37 (± 5.04)	< 0.001	24.98 (± 2.96)	32.15 (± 4.94)	< 0.001
BMI, kg/m^2^ < 25 25 to < 30 ≥ 30	439 (51.35) 378 (44.21) 38 (4.44)	9 (1.92) 160 (34.12) 300 (63.97)	< 0.001	440 (51.40) 378 (44.16) 38 (4.44)	14 (2.38) 206 (35.03) 368 (62.59)	< 0.001
Waist circumference, cm	86.21 (± 9.69)	106.98 (± 10.18)	< 0.001	86.22 (± 9.68)	107.00 (± 10.08)	< 0.001
Elevated waist circumference	201 (23.51)	400 (85.29)	< 0.001	201 (23.48)	503 (85.54)	< 0.001
Hypertension	398 (46.60)	333 (71.00)	< 0.001	398 (46.55)	427 (72.62)	< 0.001
Dyslipidaemia	467 (54.62)	372 (79.32)	< 0.001	468 (54.67)	461 (78.40)	< 0.001
Type 2 diabetes mellitus	30 (3.51)	70 (14.93)	< 0.001	30 (3.50)	88 (14.97)	< 0.001
Metabolic syndrome	110 (12.88)	296 (63.11)	< 0.001	110 (12.87)	375 (63.78)	< 0.001
Prevalent MACE	51 (5.96)	56 (11.94)	< 0.001	51 (5.96)	69 (11.73)	< 0.001
Prevalent atrial fibrillation	8 (0.94)	4 (0.85)	1.00	8 (0.93)	5 (0.85)	0.868
Lipid-lowering medication	44 (5.15)	37 (7.89)	0.046	44 (5.14)	49 (8.33)	0.015
Diabetes medication	22/843 (2.61)	45/466 (9.66)	< 0.001	22/844 (2.61)	52/584 (8.90)	< 0.001
Hypertension medication	136/849 (16.02)	161/468 (34.40)	< 0.001	136/850 (16.00)	199/586 (33.96)	< 0.001
Aspirin	84/847 (9.92)	61/466 (13.09)	0.079	84/848 (9.91)	74/584 (12.67)	0.101
Excessive alcohol consumption	148 (17.31)	0 (0)	< 0.001	147 (17.17)	97 (16.50)	0.736
Viral hepatitis	26 (3.04)	0 (0)	< 0.001	26 (3.04)	25 (4.25)	0.219
Smoking status Non-smoker Ex-smoker Current	456 (53.52) 242 (28.40) 154 (18.08)	217 (46.37) 180 (38.46) 71 (15.17)	< 0.001	456 (53.46) 243 (28.49) 154 (18.05)	246 (41.91) 240 (40.89) 101 (17.21)	< 0.001
Adequate diet	188/852 (22.07)	114/469 (24.31)	0.353	188/853 (22.04)	143/587 (24.36)	0.304
Fasting glucose, mmol/L	5.1 (± 0.9)	5.7 (± 1.5)	< 0.001	5.1 (± 0.9)	5.8 (± 1.6)	< 0.001
HbA1c, %	5.2 (± 0.4)	5.5 (± 0.7)	< 0.001	5.2 (± 0.4)	5.5 (± 0.7)	< 0.001
Total cholesterol, mmol/L	5.2 (± 0.9)	5.4 (± 1.1)	0.014	5.2 (± 0.9)	5.4 (± 1.1)	< 0.001
LDL-C, mmol/L	3.2 (± 0.8)	3.2 (± 0.9)	0.117	3.2 (± 0.8)	3.3 (± 0.9)	0.037
HDL-C, mmol/L	1.6 (± 0.4)	1.2 (± 0.3)	< 0.001	1.6 (± 0.4)	1.2 (± 0.3)	< 0.001
Low HDL	153 (17.89)	204 (43.50)	< 0.001	153 (17.87)	243 (41.33)	< 0.001
Triglycerides, mmol/L	1.0 (0.7–1.3)	1.7 (1.3–2.4)	< 0.001	1.0 (0.7–1.3)	1.7 (1.3–2.4)	< 0.001
Elevated triglycerides	124 (14.50)	270 (57.57)	< 0.001	125 (14.60)	341 (57.99)	< 0.001
Linked	660 (77.19)	390 (83.16)	0.010	660 (77.10)	491 (83.50)	0.003

Continuous variables presented as mean (± standard deviation) or median (interquartile range); categorical variables presented as frequency (%)

*NAFLD* non-alcoholic fatty liver disease; *MAFLD* metabolic-(dysfunction)-associated fatty liver disease; *BMI* body mass index; *MACE* major adverse cardiovascular events; *HbA1c* glycosylated hemoglobin A1c; *LDL-C* low-density lipoprotein cholesterol; *HDL-C* high-density lipoprotein cholesterol

During the follow-up period, 169/1324 (12.8%) developed 3-point MACE, 249/1324 (18.8%) developed 5-point MACE, 81/1324 (6.1%) died from CVD, and 125/1324 (9.4%) developed AF in the NAFLD analysis. During the MAFLD follow-up period, 192/1444 (13.3%) developed 3-point MACE, 281/1444 (19.5%) developed 5-point MACE, 89/1444 (6.2%) died from CVD, and 145/1444 (10.0%) developed AF.

### Primary outcome

Follow-up time for 3-point MACE was 23,577 and 25,469 person-years for NAFLD and MAFLD, respectively. Crude 3-point MACE incidence rates were 9.81 per 1000 person-years (95% CI 7.88–12.21) vs 5.77 per 1000 person-years (95% CI 4.69–7.11), and 10.27 per 1000 person-years (95% CI 8.47–12.46) vs. 5.76 per 1000 person-years (95% CI 4.68–7.10) for those with vs without NAFLD, and with vs without MAFLD, respectively. The IRRs were 1.70 (95% CI 1.24–2.32) and 1.78 (95% CI 1.33–2.39) respectively for NAFLD and MAFLD. Both forms of FLD were predictors of 3-point MACE on univariate analysis and on multiple models adjusting for relevant demographic and lifestyle factors (Table 2). On fully adjusted models (Table 2 and Supplementary Table 2) controlling for metabolic risk factors and baseline prevalent MACE, both forms of FLD independently predicted 3-point MACE (NAFLD: sHR 1.56 [95% CI 1.12–2.19]; MAFLD: sHR 1.51 [95% CI 1.11–2.06]). The effect size was similar for each form of FLD.

**Table 2:** Cox proportional hazards regression evaluating association between fatty liver disease and 3-point major adverse cardiovascular events (MACE)

	NAFLD	MAFLD
Univariate	1.70 (1.26–2.30)	1.76 (1.33–2.34)
Model 1	1.59 (1.16–2.17)	1.57 (1.17–2.10)
Model 2	1.67 (1.21–2.30)	1.64 (1.22–2.21)
Model 3	1.56 (1.12–2.19)	1.51 (1.11–2.06)

Data presented as sub-hazard ratios (sHR) with 95% confidence intervals

*NAFLD* non-alcoholic fatty liver disease, *MAFLD* metabolic-(dysfunction) associated fatty liver disease

Model 1 = fatty liver disease, age, gender and education

Model 2 = Model 1 + smoking status and diet adequacy

Model 3 = Model 2 + baseline MACE, type 2 diabetes mellitus, hypertension and dyslipidaemia

### Secondary outcomes

#### CVD-related death

Over 24,112 and 26,111 person-years follow-up time for NAFLD and MAFLD, crude incidence rates of CVD-related death were 4.15 per 1000 person-years (95% CI 2.98–5.78) vs 2.93 per 1000 person-years (95% CI 2.20–3.92), and 4.13 per 1000 person-years (95% CI 3.06–5.57) vs 2.93 per 1000 person-years (95% CI 2.19–3.91) for those with vs without NAFLD, and with vs without MAFLD, respectively (NAFLD MRR 1.41 [95% CI 0.88–2.24]; MAFLD MRR 1.41 [95% CI 0.91–2.18]). Neither form of FLD was a predictor for CVD death, either on univariate or multivariate analyses (Table 3).

**Table 3:** Cox proportional hazards regression evaluating association between fatty liver disease and cardiovascular disease-related death and 5-point major adverse cardiovascular events (MACE)

	NAFLD			MAFLD		
	CVD death	5-point MACE	Atrial fibrillation*	CVD death	5-point MACE	Atrial fibrillation*
Univariate	1.40 (0.90–2.18)	1.49 (1.16–1.92)	1.45 (1.02–2.08)	1.38 (0.91–2.09)	1.56 (1.23–1.97)	1.59 (1.15–2.21)
Model 1	1.40 (0.88–2.21)	1.35 (1.04–1.75)	1.24 (0.85–1.82)	1.32 (0.86–2.04)	1.35 (1.06–1.73)	1.31 (0.92–1.85)
Model 2	1.44 (0.89–2.32)	1.40 (1.08–1.83)	1.20 (0.81–1.79)	1.35 (0.87–2.11)	1.41 (1.10–1.80)	1.32 (0.93–1.88)
Model 3	1.36 (0.80–2.30)	1.29 (0.97–1.71)	1.13 (0.73–1.75)	1.32 (0.81–2.12)	1.28 (0.99–1.66)	1.15 (0.79–1.67)

Data presented as sub-hazard ratios (sHR) with 95% confidence intervals

*NAFLD* non-alcoholic fatty liver disease, *MAFLD* metabolic-(dysfunction) associated fatty liver disease, *CVD* cardiovascular disease, *MACE*, major adverse cardiovascular events

Model 1 = fatty liver disease, age, gender and education

Model 2 = Model 1 + smoking status and diet adequacy

Model 3 = Model 2 + baseline MACE, type 2 diabetes mellitus, hypertension and dyslipidaemia

^*^Atrial fibrillation Models were the same as above except Model 2 also adjusted for excessive alcohol consumption and Model 3 also adjusted for excessive alcohol consumption and baseline atrial fibrillation

Ischaemic heart disease was the main cause of CVD-related death in both forms of FLD (NAFLD: 16/35 [45.7%], MAFLD: 20/43 [46.5%]), with a smaller number related to cerebrovascular disease (NAFLD: 7/35 [20%], MAFLD: 8/43 [18.5%]) and the rest from other CVD-related causes.

#### 5-point MACE

Crude incidence rates for 5-point MACE for participants with NAFLD, no NAFLD, MAFLD and no MAFLD were 13.80 per 1000 person-years (95% CI 11.44–16.65), 9.25 per 1000 person-years (95% CI 7.84–10.92), 14.54 per 1000 person-years (95% CI 12.33–17.15) and 9.24 per 1000 person-years (95% CI 7.83–10.90), respectively (NAFLD IRR: 1.49 [95% CI 1.15–1.93]; MAFLD IRR: 1.57 [95% CI 1.24–2.00]). Moreover, when testing the association between FLD and 5-point MACE, the findings were similar to the primary outcome, with both forms of FLD conferring a higher risk of 5-point MACE on univariate and multivariate analysis adjusted for baseline demographics and lifestyle factors, with a trend to significance for both forms of FLD on the fully adjusted model accounting for metabolic risk factors (Table 3). Complete results including all covariates in fully adjusted Model 3 are provided in Supplementary Table 2.

#### Sensitivity analyses

On sensitivity analysis, excluding participants with baseline prevalent MACE, the results held true as per the primary analysis for MAFLD and to a lesser degree NAFLD, albeit with attenuation of the effect size (Supplementary Table 3). Once more, when comparing those with FLD to those with definitively no steatosis (i.e., FLI < 30), the results held true for 3-point MACE on the fully adjusted models for NAFLD and to a lesser degree MAFLD (Supplementary Table 4). When only considering primary reason for hospitalization for defining longitudinal outcomes, the magnitude of effect for 3-point MACE was marginally reduced, whilst it was accentuated for 5-point MACE such that the result was significant for both NAFLD and MAFLD. On subgroup analysis, NAFLD and MAFLD participants with normal ALT were at a heightened risk of 3-point MACE, whilst there was no significant difference for 3-point MACE in those with elevated ALT. No subgroup was associated with increased 5-point MACE or CVD death when stratified according to normal or elevated ALT (Supplementary Table 5).

#### CVD during follow-up

Over the follow-up period, incident MI occurred in 64/1217 (5.3%), incident CVA in 45/1217 (3.7%), incident CCF in 81/1217 (6.7%) and incident UA in 42/1217 (3.5%) in the NAFLD analysis, whilst the rates in the MAFLD analysis were 78/1324 (5.9%) for incident MI, 49/1324 (3.7%) for incident CVA, 83/1324 (7.0%) for incident CCF and 50/1324 (3.8%) for incident UA. Considering incident non-fatal CVD events individually, MI and CCF were the most common to occur in both forms of FLD (MI—NAFLD: 4.87 per 1000 person-years [95% CI 3.57–6.63], MAFLD: 5.11 per 1000 person-years [95% CI 3.84–6.79]; CCF—NAFLD: 6.87 per 1000 person-years [5.28–8.92], MAFLD: 5.21 per 1000 person-years [3.93–6.92]), followed by CVA and lastly UA (Table 4). The proportion of events amongst each FLD was similar.

**Table 4:** Incident non-fatal cardiovascular events in fatty liver disease participants without prevalent major adverse cardiovascular events at baseline

	NAFLD (*n* = 413)	MAFLD (*n* = 519)
Myocardial infarction	4.87 (3.57–6.63)	5.11 (3.84–6.79)
Cerebrovascular accident	2.75 (1.83–4.14)	2.67 (1.81–3.96)
Congestive cardiac failure	6.87 (5.28–8.92)	5.21 (3.93–6.92)
Unstable angina	2.30 (1.37–3.60)	2.16 (1.40–3.35)

Data presented as incidence rate per 1000 person-years (95% confidence interval)

*NAFLD* non-alcoholic fatty liver disease, *MAFLD* metabolic-(dysfunction) associated fatty liver disease

#### Atrial fibrillation

Incidence rates for atrial fibrillation were 6.73 per 1000 person-years (95% CI 5.17–8.77) vs 4.57 per 1000 person-years (95% CI 3.62–5.78), and 7.47 per 1000 person-years (95% CI 5.96–9.37) vs 4.57 per 1000 person-years (95% CI 3.61–5.77) for those with vs without NAFLD, and those with vs without MAFLD; NAFLD IRR: 1.47 [95% CI 1.01–2.13], MAFLD IRR: 1.64 [95% CI 1.17–2.30]). Neither NAFLD nor MAFLD was associated with a higher risk of AF on any multivariable model (Table 3 and Supplementary Tables 3–5).

## Discussion

To date, the influence of FLD—whether NAFLD or MAFLD—on CVD outcomes in Australia has not been established. In this seminal study of a randomly sampled cohort of adults from a major regional center in Australia with over 20 years of follow-up time, we have demonstrated that both forms of FLD carry about a 50% increased risk of 3-point MACE after correction for known CVD risk factors and have a similar influence over 3-point MACE as each other. Almost half the CVD-related deaths in participants with FLD were due to IHD, and non-fatal MI incidence was more common than incident CVA.

FLD and CVD have shared pathophysiology related to the MetSyn, obesity and insulin resistance. Putative factors implicating FLD as an independent risk factor for CVD include a systemic pro-inflammatory state, oxidative stress, abnormal lipid metabolism and direct atherogenicity linked to steatohepatitis itself [24, 25]. Unlike traditional cardiometabolic risk factors of hypertension, dyslipidemia and T2DM, there is currently no approved pharmacotherapy for FLD available across the Asia–Pacific region, which has implications for how modifiable a factor FLD is when considering global CVD risk.

Along with extrahepatic malignancy, CVD has consistently been demonstrated to be the foremost cause of death amongst people with FLD [2, 3]. Meta-analyses investigating the association of NAFLD with CVD have established NAFLD to independently increase the risk of non-fatal CVD, but the impact on fatal CVD has drawn contrasting results [9–11]. As such, our finding that NAFLD increases the risk of 3-point MACE—a combined outcome of non-fatal and fatal CVD—but not fatal CVD, and with a trend toward significance for 5-point MACE, is in keeping with the existing literature. It may be that the current study was insufficiently powered to detect differences between groups for fatal CVD. Notably, published meta-analyses are under-represented by studies from Oceania, with no study included from this region, but rather include studies primarily originating from the US, Europe and Asia. This is relevant given the differences in ethnic, cultural, and environmental factors, as well as access to health care, between geographic regions, which could impact the outcome.

Since the FLD nomenclature has shifted, there has been interest in whether the altered diagnostic criteria could have a bearing on clinical outcomes. This is particularly relevant to the proposed change from NAFLD to MAFLD, with the latter allowing for additional aetiologies of chronic liver disease. In the current study, the association between NAFLD and MAFLD and CVD outcomes was near identical, whether investigating 3- or 5-point MACE, or CVD death. The prevailing published reports have not reliably established a difference between the varying forms of FLD, with one large study from Korea suggesting MAFLD but not NAFLD to increase CVD death [26]; however this not being borne out in studies from the US [27, 28]. Trichotomizing FLD to those who meet both NAFLD and MAFLD diagnoses and comparing them to those with MAFLD-only and NAFLD-only, appears to signal differences between groups; MAFLD-only participants carry the highest risk, whilst NAFLD-only participants have the lowest risk that is comparable to the non-FLD population [26, 29, 30]. However, this finding is inconclusive, with other authors finding no difference between groups [27, 28, 31]. We were unable to stratify participants from our cohort in the same manner, as all but a single NAFLD participant met the MAFLD diagnosis. As such, this warrants further investigation, particularly given the complex association between alcohol consumption and CVD [32].

Whilst this is the first description of the association between various forms of FLD and CVD outcomes in a well-characterized randomly sampled cohort of adults from Australia with a long follow-up time, there are limitations to the current study. The cohort was from a single regional area in Australia, which impacts the generalizability of the results to metropolitan locations. However, the concordance in results of the current study with the literature from overseas cohorts with disparate ethnic and socioeconomic backgrounds, increases the external validity of the result. Second, although physical activity—a known lifestyle factor impacting CVD outcomes—was measured in the current cohort at baseline, the data were missing for ~ 30% of participants. Given there was no detectable difference between the FLD and non-FLD groups in amount of physical activity participation per week, this was excluded from the multivariate models. Given BMI and waist circumference are integral in calculating FLI, these too were omitted from multivariable models. Neither homeostatic model assessment for insulin resistance nor high-sensitivity C-reactive protein were measured in participants, which form part of the diagnostic criteria for MAFLD, however, this is unlikely to have significantly influenced the result. Further, the predominant sampled participants for this study are likely to represent a healthy bias. Finally, inherent to all data linkage studies, there is a risk of misattribution/miscoding or missing outcomes, including if participants moved outside the state of Victoria during follow-up.

In conclusion, both NAFLD and MAFLD are associated with a 50% increased risk of 3-point MACE, independent of well-recognized cardiometabolic risk factors. Public health programs are required to ensure that FLD is considered when establishing individuals’ CVD risk profile and CVD outcomes need to be explored when investigating the efficacy of novel FLD pharmacotherapeutic agents.

### Supplementary Information

Below is the link to the electronic supplementary material.

Supplementary file1 (DOCX 25 kb)

## Data Availability

Data may be available upon reasonable request to the corresponding author and following approval by the Victorian Department of Health for outcome data, given linked data is stored on a secure virtual network.
