# Supplementary material for: NAFLD and MAFLD independently increase the risk of major adverse cardiovascular events (MACE): a 20-year longitudinal follow-up study from regional Australia
Source: Hepatol Int. 2024 Jul 15;18(4):1135–43. doi: 10.1007/s12072-024-10706-1 (PMC11297804; doi:10.1007/s12072-024-10706-1)
Supplement: Supplementary file 1 — Supplementary file1 (DOCX 25 kb) [file 12072_2024_10706_MOESM1_ESM.docx]

**Supplementary Appendix**

**Supplementary Table 1.** ICD-10 codes utilized to define cardiovascular events

| **Outcome** | **ICD-10 codes** |
| --- | --- |
| Atrial fibrillation | I48 |
| Myocardial infarction | I21, I22, I24 |
| Cerebrovascular accident (incidence) | I60-I64 |
| Congestive cardiac failure | I11.0, I25.5, I42, I43, I50 |
| Unstable angina | I20.0 |
| CVD-related death | I00-I73 |
| Ischaemic heart disease-related death | I20-25 |
| Cerebrovascular disease-related death | I60-I69 |
| ICD-10 = International Statistical Classification of Diseases and Related Health Problems, 10^th^ revision | |

**Supplementary Table 2.** Complete results for all covariates in Model 3 of Cox proportional hazards regression multivariate model for CVD death, major adverse cardiovascular outcome (MACE) and atrial fibrillation

|  | **NAFLD** | | | | **MAFLD** | | | |
| --- | --- | --- | --- | --- | --- | --- | --- | --- |
|  | **3-point MACE** | **5-point MACE** | **CVD death** | **Atrial fibrillation** | **3-point MACE** | **5-point MACE** | **CVD death** | **Atrial fibrillation** |
| Fatty liver disease | 1.56  (1.12-2.19) | 1.29  (0.97-1.71) | 1.36  (0.80-2.30) | 1.13  (0.73-1.75) | 1.51  (1.11-2.06) | 1.28  (0.99-1.66) | 1.31  (0.81-2.12) | 1.15  (0.79-1.67) |
| Age | 1.06  (1.05-1.07) | 1.05  (1.04-1.06) | 1.10  (1.08-1.12) | 1.04  (1.03-1.06) | 1.06  (1.05-1.07) | 1.05  (1.04-1.06) | 1.10  (1.08-1.12) | 1.04  (1.03-1.05) |
| Female gender | 0.78  (0.56-1.09) | 0.68  (0.52-0.89) | 0.78  (0.46-1.30) | 0.75  (0.50-1.13) | 0.73  (0.54-1.00) | 0.67  (0.52-0.87) | 0.71  (0.44-1.14) | 0.75 |
| Education (secondary school and above) | 1.02  (0.73-1.43) | 0.97  (0.74-1.28) | 1.08  (0.67-1.76) | 0.87  (0.58-1.29) | 1.07  (0.78-1.46) | 0.99  (0.77-1.29) | 1.03  (0.65-1.65) | 0.83  (0.57-1.21) |
| Smoking status  Non-smoker  Ex-smoker  Current smoker | Reference  1.16  (0.71-1.91)  0.79  (0.54-1.14) | Reference  1.14  (0.76-1.72)  0.76  (0.56-1.04) | Reference  0.93  (0.39-2.19)  0.74  (0.42-1.29) | Reference  1.00  (0.56-1.79)  0.83  (0.53-1.28) | Reference  1.22  (0.78-1.93)  0.78  (0.56-1.09) | Reference  1.16  (0.80-1.70)  0.76  (0.57-1.00) | Reference  0.90  (0.40-2.04)  0.74  (0.44-1.24) | Reference  0.97  (0.56-1.70)  1.06  (0.72-1.56) |
| Healthy diet | 0.79  (0.54-1.16) | 0.90  (0.66-1.23) | 1.02  (0.60-1.72) | 0.88  (0.56-1.37) | 0.86  (0.61-1.23) | 0.93  (0.69-1.24) | 1.08  (0.66-1.76) | 0.84  (0.56-1.27) |
| Alcohol excess | - | - | - | 0.76  (0.37-1.59) | - | - | - | 1.04  (0.66-1.64) |
| Type 2 diabetes mellitus | 0.49  (0.27-0.88) | 0.68  (0.42-1.09) | 0.47  (0.22-1.01) | 1.10  (0.62-1.93) | 0.53  (0.31-0.90) | 0.69  (0.45-1.06) | 0.43  (0.21-0.92) | 1.26  (0.77-2.08) |
| Hypertension | 1.39  (0.91-2.13) | 1.28  (0.90-1.82) | 1.48  (0.69-3.19) | 1.39  (0.85-2.26) | 1.61  (1.08-2.42) | 1.43  (1.03-2.00) | 1.71  (0.82-3.55) | 1.58  (0.99-2.52) |
| Dyslipidaemia | 1.65  (1.13-2.42) | 1.52  (1.12-2.06) | 1.57  (0.88-2.82) | 1.11  (0.74-1.67) | 1.70  (1.19-2.44) | 1.57  (1.18-2.10) | 1.50  (0.87-2.58) | 1.31  (0.89-1.92) |
| Baseline prevalent MACE | 1.48  (0.94-2.32) | 1.82  (1.24-2.65) | 1.84  (1.03-3.30) | 1.05  (0.57-1.92) | 1.41  (0.92-2.15) | 1.74  (1.22-2.47) | 1.75  (1.00-3.05) | 0.97  (0.55-1.70) |
| Baseline prevalent atrial fibrillation | - | - | - | 6.79  (2.17-21.26) | - | - | - | 5.28  (1.83-15.27) |
| Data presented as sub-hazard ratios (sHR) with 95% confidence intervals  NAFLD = non-alcoholic fatty liver disease; MAFLD = metabolic-(dysfunction) associated fatty liver disease; MACE = major adverse cardiovascular events | | | | | | | | |

**Supplementary Table 3.** Sensitivity analysis for cardiovascular disease related outcomes, excluding participants with baseline prevalent major adverse cardiovascular events (MACE) or baseline prevalent atrial fibrillation

|  | **NAFLD** | | | | **MAFLD** | | | |
| --- | --- | --- | --- | --- | --- | --- | --- | --- |
|  | **3-point MACE** | **5-point MACE** | **CVD death** | **Atrial fibrillation*** | **3-point MACE** | **5-point MACE** | **CVD death** | **Atrial fibrillation*** |
| Univariate | 1.63  (1.16-2.29) | 1.36  (1.03-1.81) | 1.23  (0.72-2.10) | 1.36  (0.84-1.90) | 1.74  (1.27-2.39) | 1.48  (1.14-1.92) | 1.30  (0.79-2.12) | 1.50  (1.04-2.16) |
| Model 1 | 1.54  (1.08-2.19) | 1.26  (0.94-1.69) | 1.34  (0.76-2.35) | 1.07  (0.69-1.68) | 1.55  (1.12-2.15) | 1.29  (0.99-1.69) | 1.29  (0.77-2.15) | 1.20  (0.81-1.78) |
| Model 2 | 1.64  (1.14-2.36) | 1.32  (0.98-1.77) | 1.41  (0.78-2.56) | 1.08  (0.67-1.72) | 1.65  (1.18-2.32) | 1.36  (1.04-1.79) | 1.36  (0.80-2.33) | 1.22  (0.82-1.81) |
| Model 3 | 1.43  (0.99-2.07) | 1.16  (0.85-1.58) | 1.27  (0.67-2.40) | 0.96  (0.58-1.59) | 1.43  (1.02-2.01) | 1.21  (0.91-1.60) | 1.23  (0.69-2.19) | 1.03  (0.67-1.56) |
| Data presented as sub-hazard ratios (sHR) with 95% confidence intervals  NAFLD = non-alcoholic fatty liver disease; MAFLD = metabolic-(dysfunction) associated fatty liver disease; CVD – cardiovascular disease; MACE = major adverse cardiovascular events  Model 1 = fatty liver disease, age, gender and education  Model 2 = Model 1 + smoking status and diet adequacy  Model 3 = Model 2 + type 2 diabetes mellitus, hypertension and dyslipidaemia  *Atrial fibrillation Models were the same as above except Model 2 and Model 3 also adjusted for excessive alcohol consumption | | | | | | | | |

**Supplementary Table 4.** Sensitivity analysis for cardiovascular disease related outcomes, comparing participants with fatty liver disease (FLD) with those with definitively no FLD (ie. Fatty Liver Index <30) and when only considering primary reason for hospitalization

|  | **NAFLD** | | | | **MAFLD** | | | |
| --- | --- | --- | --- | --- | --- | --- | --- | --- |
|  | **3-point MACE** | **5-point MACE** | **CVD death** | **Atrial fibrillation*** | **3-point MACE** | **5-point MACE** | **CVD death** | **Atrial fibrillation*** |
| Model 3 (FLI <30) | 1.61  (1.03-2.52) | 1.37  (0.94-1.98) | 1.42  (0.68-2.96) | 1.08  (0.65-1.81) | 1.50  (0.99-2.25) | 1.31  (0.94-1.84) | 1.29  (0.67-2.49) | 1.08  (0.68-1.71) |
| Model 3  (primary reason hospitalization) | 1.52  (1.07-2.15) | 1.40  (1.03-1.89) | N/A | 1.00  (0.57-1.77) | 1.49  (1.09-2.05) | 1.33  (1.01-1.76) | N/A | 0.95  (0.57-1.57) |
| Data presented as sub-hazard ratios (sHR) with 95% confidence intervals  NAFLD = non-alcoholic fatty liver disease; MAFLD = metabolic-(dysfunction) associated fatty liver disease; CVD – cardiovascular disease; MACE = major adverse cardiovascular events; FLI = fatty liver index  Model 3 = fatty liver disease, age, gender, education, smoking status, diet adequacy, baseline MACE, type 2 diabetes mellitus, hypertension and dyslipidaemia  *Atrial fibrillation Model also adjusted for excessive alcohol consumption and baseline atrial fibrillation | | | | | | | | |

**Supplementary Table 5.** Subgroup analysis for cardiovascular disease related outcomes, comparing participants with fatty liver disease (FLD) with and without elevated ALT

|  | **NAFLD** | | | | **MAFLD** | | | |
| --- | --- | --- | --- | --- | --- | --- | --- | --- |
|  | **3-point MACE** | **5-point MACE** | **CVD death** | **Atrial fibrillation*** | **3-point MACE** | **5-point MACE** | **CVD death** | **Atrial fibrillation*** |
| Model 3 (normal ALT) | 1.54  (1.07-2.20) | 1.32  (0.98-1.78) | 1.41  (0.81-2.43) | 1.22  (0.78-1.92) | 1.51  (1.09-2.09) | 1.32  (1.00-1.73) | 1.34  (0.81-2.21) | 1.24  (0.84-1.84) |
| Model 3 (elevated ALT) | 1.69  (0.96-2.98) | 1.13  (0.69-1.87) | 1.06  (0.36-3.14) | 0.68  (0.29-1.58) | 1.53  (0.93-2.51) | 1.14  (0.74-1.75) | 1.14  (0.46-2.84) | 0.81  (0.43-1.52) |
| Data presented as sub-hazard ratios (sHR) with 95% confidence intervals  NAFLD = non-alcoholic fatty liver disease; MAFLD = metabolic-(dysfunction) associated fatty liver disease; CVD – cardiovascular disease; MACE = major adverse cardiovascular events; ALT = alanine aminotransferase  Model 3 = fatty liver disease, age, gender, education, smoking status, diet adequacy, baseline MACE, type 2 diabetes mellitus, hypertension and dyslipidaemia  *Atrial fibrillation Model also adjusted for excessive alcohol consumption and baseline atrial fibrillation | | | | | | | | |
